# Outcomes of COVID‐19 in patients with lymphomas participating in registered clinical trials: A real‐world study from China in the Omicron outbreak era

**DOI:** 10.1002/cam4.6678

**Published:** 2023-11-27

**Authors:** Peng Sun, Hang Yang, Baitian Zhao, Yu Wang, Man Nie, Kangming Huang, Zhiming Li

**Affiliations:** ^1^ Department of Medical Oncology Sun Yat‐Sen University Cancer Center Guangzhou China; ^2^ State Key Laboratory of Oncology in South China Guangdong Provincial Clinical Research Center for Cancer Guangzhou China; ^3^ Department of Clinical Trials Center Sun Yat‐Sen University Cancer Center Guangzhou China

**Keywords:** clinical trials, COVID‐19, lymphoma

## Abstract

**Background:**

This real‐world study investigated the outcome of COVID‐19 in lymphoma patients participating in registered clinical trials and explored potential risk factors with the outcome of COVID‐19 during the first wave of the Omicron outbreak in China.

**Methods:**

One hundred and ten patients participating in registered clinical trials and diagnosed with COVID‐19 in our center between December 1, 2022, and January 31, 2023, were included.

**Results:**

Four (3.6%) patients were identified as severe COVID‐19 and 2 (1.8%) as critical COVID‐19, respectively. The mortality rate observed was 2.73% for the entire cohort, 33.3% for the severe/critical COVID‐19 group, and 18.8% for the hospitalized group. The 90‐day OS was 98.2% for the entire cohort, 66.7% for the severe/critical COVID‐19 group, and 87.5% for the hospitalized group. Advanced age (≥70 years), comorbidities, and PI3K inhibitor‐containing regimen were significantly associated with the severity of COVID‐19. Patients with indolent B‐cell non‐Hodgkin lymphomas were less likely to be hospitalized for COVID‐19.

**Conclusion:**

This study reported similar clinical features of COVID‐19 in our cohort with that of non‐hematological malignancy (HM) patients, while the proportion of severe/critical COVID‐19 and the mortality rate were relatively higher than non‐HM patients. Our findings provided valuable experience to aid clinical researchers with managing lymphoma patients participating in registered clinical trials during the ongoing pandemic of the Omicron variant.

## 1 INTRODUCTION

Since the outbreak in Wuhan, China, in 2019,^1^, ^2^, ^3^ COVID‐19, caused by the severe acute respiratory syndrome coronavirus 2 (SARS‐CoV‐2), has become a global health event for the past 3 years. Current studies have demonstrated that patients with malignancies were more vulnerable to COVID‐19.^4^, ^5^, ^6^ The probability of severe COVID‐19 in patients with malignancies was reported to be 30% to 40%, which manifested in admission to an intensive care unit, the need for mechanical ventilation, and even death. In comparison, the proportion of non‐cancer patients developing severe COVID‐19 was less than 10%.^7^, ^8^ Compared with patients with solid cancers, patients with hematological malignancies (HM), such as leukemias, lymphomas, and multiple myelomas, usually have long‐lasting immunodeficiency due to the malignancy itself, immunosuppressive treatments, or as a consequence of procedures such as hematopoietic stem‐cell transplantation, would more likely to suffer severe/critical COVID‐19.^9^, ^10^, ^11^

China changed its public health policy in containing COVID‐19 in December 2022.^12^ Subsequently, a large‐scale outbreak of SARS‐CoV‐2 infections occurred in the entire country, mainly caused by the Omicron subvariants BF.7 and BA.5.2.^12^ Compared with the initial strain in 2019, Omicron is more infectious and less virulent, and the clinical manifestations vary in severity after infection, with the majority showing flu‐like symptoms.^13^ The first wave of COVID‐19 brought a challenge to Chinese patients with HM. Zhu et al. conducted a retrospective, observational study of COVID‐19 patients with HMs in Beijing in the latest Omicron wave. They demonstrated that the rate of severe/critical COVID‐19 was 5.3%, and the mortality rate was 2.2%,^14^ much lower than that of the HM patient population reported in the early stage of the COVID‐19 pandemic.^15^ Till now, only a few studies reported the impact of the Omicron variant of SARS‐CoV‐2 on the clinical outcome of HM patients.

Malignant lymphoma is a heterogeneous group of HMs, accompanied by multiple immune dysfunctions of the innate and adaptive immune system and relatively low immunoglobulin serum levels. Lymphoma patients were also considered vulnerable to severe COVID‐19 infection.^16^, ^17^ Lymphoma cases have been generally under‐represented in most studies of cancer or HM series, with limited reports addressing the outcome of COVID‐19. Available data regarding this issue were mainly from hospitalized patients and focused on the first wave of COVID‐19 infection, when the Omicron variant was not the primary epidemic strain.

Although modern therapeutic approaches such as monoclonal antibodies, immunotherapy, and tyrosine kinase inhibitors have substantially prolonged the survival of lymphoma patients, clinical trials are essential for advancing treatment and providing access to novel and potentially effective agents for lymphoma patients. Over 40% of hospitalized lymphoma patients in our center participated in clinical trials. Compared with those receiving routine treatment, lymphoma patients participating in registered clinical trials often have better compliance and can provide more complete medical records and more detailed information on COVID‐19. Therefore, participants in registered clinical trials were ideal candidates for the research of lymphoma and COVID‐19. Herein, we conducted this real‐world study to investigate the outcome of COVID‐19 in lymphoma patients participating in registered clinical trials and to explore potential risk factors with the outcome of COVID‐19 during the first wave of the Omicron outbreak in China.

## 2 METHODS

### 2.1 Study design

This single‐center, real‐world study of COVID‐19 included a retrospective data review. All lymphoma patients participating in registered clinical trials between December 1, 2022, and January 31, 2023, were screened, with the data cutoff for the analyses on May 15, 2023. Inclusion criteria were as follows: (1) the diagnosis of lymphoma was confirmed by pathology according to World Health Organization criteria; (2) diagnosed with COVID‐19 based on a positive polymerase chain reaction (PCR) test result for SARS‐CoV‐2 from oropharyngeal swabs, or on a positive detection of COVID‐19 antigen from nasopharyngeal swabs and typical clinical history; (3) participated in registered clinical trials and received anti‐lymphoma treatment within 4 weeks before COVID‐19 infection; (4) complete data on clinico‐pathological characteristics, treatment approaches, and follow‐up were available. A total of 135 lymphoma patients were screened. Two patients were excluded for lost follow‐up, and 23 patients who did not report a history of COVID‐19 infection were also excluded (Figure S1). Finally, 110 cases were enrolled. Seventy‐six patients were diagnosed with COVID‐19 infection based on a positive PCR test result for SARS‐CoV‐2 from oropharyngeal swabs, and 34 patients were diagnosed on a positive detection of COVID‐19 antigen from nasopharyngeal swabs and typical clinical history. This study was approved by the Ethics Committee of Sun Yat‐Sen University Cancer Center (B2023‐075‐01) in accordance with the guiding principles of the Declaration of Helsinki and registered on ClinicalTrials.gov (NCT05827341). Written informed consent was obtained from each patient. We have uploaded the essential raw data onto the Research Data Deposit (RDDA2023450151) public platform (https://www.researchdata.org.cn).

### 2.2 Data collection

Data on patient characteristics and outcomes were extracted by investigators from medical records, and electronic survey questionnaires were performed to enrich the information. Clinico‐pathological data (age, gender, performance status, histological subtype, stage, treatments of lymphoma, phase of clinical trial, previous treatment history, disease status, blood cell parameters, and comorbidities), information on COVID‐19 (vaccination, symptoms, treatment, severity, and outcome) were recorded. The severity of COVID‐19 was evaluated according to the treatment guidelines of the National Health Commission in China (version 10.0).^18^ Comorbidities were defined as hypertension, diabetes, chronic pulmonary disease, coronary heart disease, and hepatitis B virus infection. Given the diverse and complex mechanisms of anti‐lymphoma treatment in our patients, therapeutic schemes were identified as the following categories: (1) immunotherapy, (2) biologics (single agents or in combination), (3) immunochemotherapy, and (4) other. The immunotherapy categories included treatment with monoclonal antibodies, antibody‐drug conjugates (mainly anti‐CD20 and anti‐CD30), and/or checkpoint inhibitors (most were PD‐1/PD‐L1 inhibitors); biologics included Bruton's tyrosine kinase inhibitors, PI3K inhibitors (PI3Ki), immunomodulators, BCL‐2 inhibitors, and XPO‐1 inhibitors; immunochemotherapy referred to various combinations of chemotherapy agents plus immunotherapy agents.

### 2.3 Statistical analysis

In this study, the primary outcome was the severity of COVID‐19, and the secondary outcome was the hospitalization rate for COVID‐19. Characteristics of the study population were described for the entire cohort, severe/critical COVID‐19 cohort, and hospitalized cohort. Categorical variables were summarized by frequencies and percentages, and continuous variables were described as median and range. The mortality rate and overall survival (OS) were both calculated. OS, which was defined as the time interval between the date of COVID‐19 infection and the date of death from any cause or last follow‐up, was estimated by the Kaplan–Meier method and compared by log‐rank test. Ordinal logistic regression model was used to explore the association between variables and the severity of COVID‐19, while binary logistic regression model was used to explore the association between variables and hospitalization for COVID‐19. In both models, confounding variables (gender and age) and the variables that showed statistical significance (*p* < 0.05) in univariate analysis would be further explored in multivariate analysis, and the odds ratio (OR) with 95% confidential interval (CI) was reported. Variables considered in these analyses were age (≥70 years vs. below), gender, the phase of trial (1 vs. 2/3), lymphoma status (refractory/relapsed vs. others), BMI (≥25 kg/m^2^ vs. below), baseline hemoglobulin (<12 g/dL vs. ≥12 g/dL), lymphocyte (<800/mmc vs. ≥800/mmc), neutrophil‐to‐lymphocyte ratio (>3.5 vs. ≤3.5), vaccination (no vs. yes, ≥3 doses vs. others), indolent B‐cell non‐Hodgkin's lymphoma (i‐BNHL) versus other subtypes, disease stage (advanced vs. others), international prognostic index score (≥2 vs. <2), smoking status (ever vs. never), previous treatment lines (≥3 vs. <3), previous treatment history (anti‐CD20 antibody, anti‐PD‐1/PD‐L1 antibody, and bendamustine), comorbidities (≥1 vs. none), current anti‐lymphoma approaches (PI3K inhibitors vs. others, anti‐CD20 vs. others), and the efficacy of current treatment (remission vs. others). Statistical tests were two sided, and *p*‐values < 0.05 were considered to denote statistical significance. All statistical analyses were performed by IBM SPSS 22.0 software.

## 3 RESULTS

### 3.1 Patients' characteristics

Detailed characteristics of the included patients are summarized in Table 1. Their median age was 55 years (range: 20–80 years), and 63.6% were male. There were 32 (29.1%) patients in phase 1 trials, 53 (48.2%) in phase 2 trials and 25 (22.7%) in phase 3 trials, respectively. Most of the patients were identified as relapsed/refractory disease (*n* = 98, 89.1%). Thirty‐eight patients (34.5%) received COVID‐19 vaccines, and 11 patients (10%) received no less than three doses of COVID‐19 vaccines. Lymphoma histological subtypes were diffuse large B‐cell lymphoma for 33 patients (30.0%), i‐BNHL (including follicular lymphoma, marginal zone lymphoma, chronic lymphocytic leukemia) for 45 patients (40.9%), mantle cell lymphoma (MCL) for 12 patients (10.9%), peripheral T‐cell lymphoma for 11 patients (10.0%), Hodgkin's lymphoma for seven patients (6.4%), and natural killer/T‐cell lymphoma for two patients (1.8%), respectively. Forty‐two patients (38.2%) had at least one significant comorbidity. Forty patients (36.4%) were ever smokers. According to previous treatment history, six patients (6.5%) had received ASCT, four patients (3.6%) had undergone CAR‐T therapy, 75 patients (68.2%) had received anti‐CD20 monoclonal antibodies, 16 patients (14.5%) had received anti‐PD‐1/PD‐L1 antibodies, and 13 patients (11.8%) received bendamustine. Immunotherapy was given to 36 patients (32.7%), biologics were given to 57 patients (51.8%), and immunochemotherapy was given to 14 patients (12.7%).

**TABLE 1:** Baseline characteristics of enrolled patients.

Demographic characteristics	Entire group (*n* = 110)	Severe/critical COVID‐19 (*n* = 6)	Hospitalization for COVID‐19 (*n* = 16)
Age, years			
Median (range)	55 (20–80)	57.5 (30–77)	64 (30–77)
≥70, *n* (%)	16 (14.5)	2 (33.3)	6 (37.5)
Male gender, *n* (%)	70 (63.6)	4 (66.7)	13 (81.3)
Study phase, *n* (%)			
Phase 1	32 (29.1)	2 (33.3)	4 (25.0)
Phase 2	53 (48.2)	2 (33.3)	6 (37.5)
Phase 3	25 (22.7)	2 (33.3)	6 (37.5)
Relapsed/refractory	98 (89.1)	4 (66.7)	13 (81.3)
Body mass index (kg/m^2^)			
Median (range)	23.5 (15.3–31.1)	22.6 (17.5–24.8)	22.6 (17.5–28.6)
≥25, *n* (%)	37 (33.6)	0	4 (25.0)
HB <120 g/L, *n* (%)	28 (25.5)	1 (16.7)	5 (31.3)
Lymphocyte <800/mmc, *n* (%)	10 (9.1)	0	2 (12.5)
NLR >3.5, *n* (%)	21 (19.1)	2 (33.3)	4 (25.1)
Vaccination, *n* (%)	38 (34.5)	2 (33.3)	10 (62.5)
≥3 doses of vaccination, *n* (%)	11 (10.0)	1 (16.7)	1 (6.3)
Histology subtype, *n* (%)			
DLBCL	33 (30.0)	3 (50.0)	9 (56.3)
i‐BNHL	45 (40.9)	0	1 (6.3)
MCL	12 (10.9)	2 (33.3)	3 (18.8)
PTCL	11 (10.0)	1 (16.7)	2 (12.5)
HL	7 (6.4)	0	0
NKTCL	2 (1.8)	0	1 (6.3)
Advanced stage, *n* (%)	89 (80.9)	5 (83.3)	10 (62.5)
IPI ≥2, *n* (%)	68 (61.8)	5 (83.3)	10 (62.5)
Ever smoker, *n* (%)	40 (36.4)	3 (50.0)	9 (56.3)
PTH CD20 antibody, *n* (%)	75 (68.2)	3 (50.0)	11 (68.8)
Previous treatment line ≥3, *n* (%)	44 (40.0)	3 (50.0)	9 (56.3)
CR/PR at COVID‐19, *n* (%)	78 (70.9)	4 (66.7)	8 (50.0)
PTH CART therapy, *n* (%)	4 (3.6)	1 (16.7)	2 (12.5)
PTH ASCT, *n* (%)	6 (5.5)	0	0
Comorbidity ≥1, *n* (%)	42 (38.2)	4 (66.7)	10 (62.5)
Hypertension, *n* (%)	15 (13.6)	2 (33.3)	5 (31.3)
Diabetes, *n* (%)	11 (10.0)	0	1 (6.3)
Chronic lung disease, *n* (%)	3 (2.7)	1 (16.7)	2 (12.5)
HBV infection, *n* (%)	16 (14.5)	1 (16.7)	2 (12.5)
Therapeutic schemes			
Immunotherapy	36 (32.7)	2 (33.3)	6 (37.5)
Biologics	57 (51.8)	3 (50.0)	4 (25.0)
Immunochemotherapy	14 (12.7)	1 (16.7)	4 (25.0)
Other	3 (2.7)	0	2 (12.5)
PTH bendamustine, *n* (%)	13 (11.8)	0	1 (6.3)
PTH PD‐1/PD‐L1	16 (14.5)	0	2 (12.5)

Abbreviations: ASCT, autologous stem‐cell transplantation; CART, chimeric antigen receptor T cell; CR, complete response; DLBCL, diffuse large B‐cell lymphoma; HB, hemoglobulin; HBV, hepatitis B virus; HL, Hodgkin's lymphoma; i‐BNHL, indolent B‐cell non‐Hodgkin's lymphoma; IPI, international prognostic index; MCL, mantle cell lymphoma; NKTCL, natural killer/T‐cell lymphoma; NLR, neutrophil‐to‐lymphocyte ratio; PR, partial response; PTCL, peripheral T‐cell lymphoma; PTH, previous treatment history.

### 3.2 COVID‐19‐related signs and symptoms, COVID‐19 management, and clinical outcome

**FIGURE 1:**
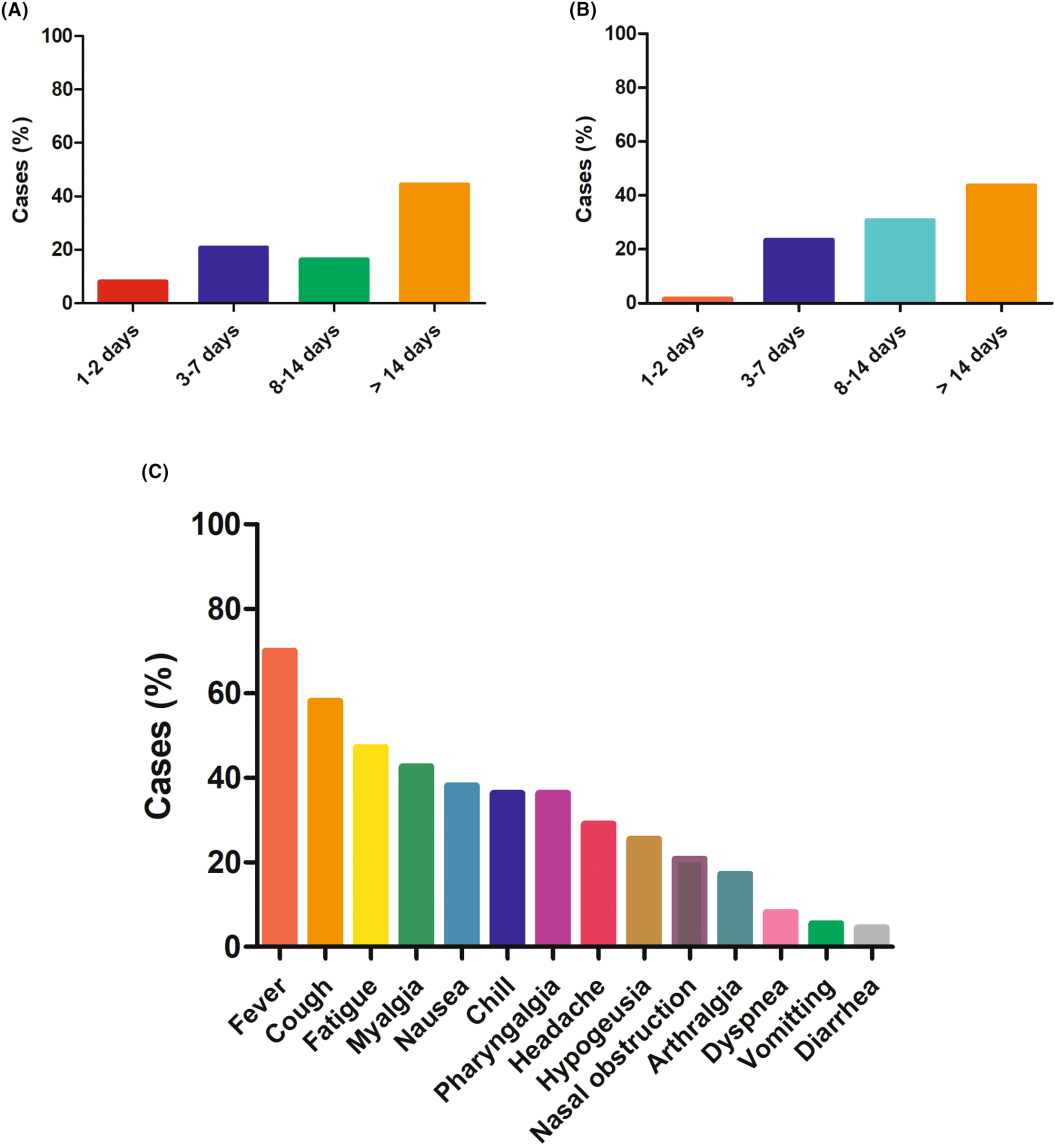
Clinical features of COVID‐19 in our patients. (A) Duration time of COVID‐19‐related symptoms. (B) Duration time of positive test of COVID‐19. (C) The spectrum of COVID‐19‐related symptoms.

Fever (70%) was the most common symptom, followed by cough (58.2%), fatigue (47.3%), myalgia (42.7%), nausea (38.2%), chill (36.4%), and pharyngalgia (36.4%), as shown in Figure 1. Thirteen patients (11.8%) received steroids and 27 (24.5%) received antibiotics. Eleven patients have been prescribed treatment against SARS‐CoV‐2: nirmatrelvir/ritonavir (PAXLOVID) was given to seven patients, either alone (*n* = 6) or associated with azvudine (*n* = 1), and azvudine was given to four patients. Duration time of COVID‐19‐related symptoms and duration time of positive test of COVID‐19 are shown in Figure 1.

**FIGURE 2:**
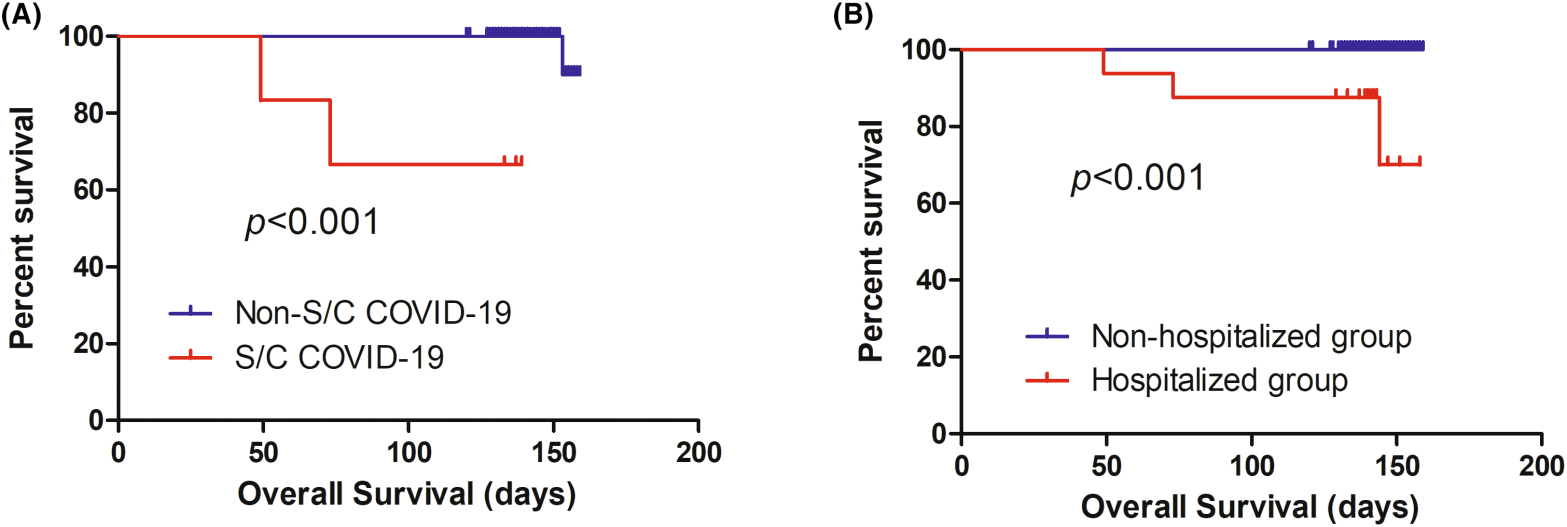
Overall survival curves for lymphoma patients with COVID‐19 (A) according to the severity of COVID‐19, and (B) according to the hospitalization for COVID‐19. S/C, severe/critical.

With a median follow‐up of 143 days from the onset of COVID‐19 (range from 49 to 159 days), the Kaplan–Meier estimate of 30‐day OS, 60‐day OS, and 90‐day OS for the entire cohort was 100%, 99.1%, and 98.2%, respectively. The 30‐day OS, 60‐day OS, and 90‐day OS was 100%, 83.3%, and 66.7% for the severe/critical COVID‐19 group, and all 100% for the mild/moderate COVID‐19 group, respectively (*p* < 0.001; Figure 2A). The 30‐day OS, 60‐day OS, and 90‐day OS was 100%, 93.8%, and 87.5% for the hospitalized group, and all 100% for the non‐hospitalized group, respectively (*p* < 0.001; Figure 2B).

According to the investigators' evaluation, 78 patients (70.9%) were identified as mild COVID‐19, 26 (23.6%) as moderate COVID‐19, four (3.6%) as severe COVID‐19, and two (1.8%) as critical COVID‐19, respectively. Ordinal logistic regression analyses of the variables associated with the severity of COVID‐19 are shown in Table 2. An age ≥70 years, phase 1 study, comorbidities, and PI3Ki‐containing regimen were all significantly associated with the severity of COVID‐19 in univariate analysis. An age ≥70 years (OR 3.531, 95% CI 1.081–11.531, *p* = 0.037), comorbidities (OR 2.659, 95% CI 1.047–6.755, *p* = 0.040), and PI3Ki‐containing regimen (OR 4.070, 95% CI 1.521–10.893, *p* = 0.005) were all significantly associated with severe/critical COVID‐19 in the multivariate analysis.

**TABLE 2:** Univariate and multivariate analyses of the severity of COVID‐19.

Variables	Univariate analysis		Multivariate analysis	
	OR (95% CI)	*p*‐value	OR (95% CI)	*p*‐value
Age ≥70 years	2.904 (1.018, 8.287)	0.046*	3.531 (1.081, 11.531)	0.037*
Gender (male vs. female)	0.878 (0.374, 2.063)	0.765	0.908 (0.354, 2.334)	0.842
Study phase (1 vs. 2/3)	2.462 (1.044, 5.805)	0.039*	1.860 (0.722, 4.789)	0.199
Relapsed/refractory	1.056 (0.263, 4.233)	0.939		
BMI ≥25 kg/m^2^	0.523 (0.210, 1.307)	0.166		
HB <12 g/L	0.754 (0.286, 1.987)	0.568		
Lymphocyte <800/mmc	0.243 (0.030, 1.993)	0.188		
NLR >3.5	1.048 (0.367, 2.993)	0.930		
Vaccination	0.442 (0.171, 1.142)	0.092		
≥3 doses of vaccination	0.241 (0.029, 1.979)	0.185		
i‐BNHL versus other	0.868 (0.379, 1.986)	0.737		
Advanced stage	0.639 (0.241, 1.695)	0.369		
IPI ≥2	0.940 (0.409, 2.158)	0.883		
Ever smoker	1.341 (0.580, 3.102)	0.493		
PTH CD20 antibody	1.449 (0.576, 3.650)	0.431		
Previous treatment line ≥3	1.079 (0.469, 2.479)	0.858		
CR/PR at COVID‐19	0.631 (0.241, 1.650)	0.348		
Comorbidity ≥1	3.481 (1.491, 8.125)	0.004*	2.659 (1.047, 6.755)	0.040*
PI3Ki‐based therapy	3.302 (1.368, 7.969)	0.008*	4.070 (1.521, 10.893)	0.005*
Anti‐CD20 therapy	0.718 (0.240, 2.143)	0.552		
PTH bendamustine	0.664 (0.172, 2.555)	0.551		
PTH PD‐1/PD‐L1	0.734 (0.221, 2.439)	0.614		

Abbreviations: ASCT, autologous stem‐cell transplantation; BMI, body mass index; CART, chimeric antigen receptor T cell; CR, complete response; HB, hemoglobulin; i‐BNHL, indolent B‐cell non‐Hodgkin's lymphoma; IPI, international prognostic index; NLR, neutrophil‐to‐lymphocyte ratio; OR, odds ratio; PI3Ki, PI3K inhibitors; PR, partial response; PTH, previous treatment history.

* *p* < 0.05.

Sixteen patients (14.5%) were admitted to hospital due to COVID‐19. Two of them died during the hospitalization (Table S1), one patient died of progressive lymphoma after recovering from moderate COVID‐19. The mortality rate observed was 2.73% (3/110, 95% CI 0%–5.8%) for the entire cohort, 33.3% (2/6, 95% CI 0%–87.5%) for the severe/critical COVID‐19 group, and 18.8% (3/16, 95% CI 0%–40.2%) for the hospitalized group. Binary logistic regression analyses of the variables associated with the hospitalization for COVID‐19 are shown in Table 3. An age ≥70 years, vaccination, i‐BNHL, and comorbidities all showed significance in univariate analysis; however, only the pathological subtype (i‐BHL vs. other) was significant in multivariate analysis. Patients with i‐BNHL tended to have a low risk of hospitalization for COVID‐19 (OR 0.113, 95% CI 0.013–0.945, *p* = 0.044).

**TABLE 3:** Univariate and multivariate analyses of the hospitalization for COVID‐19.

Variables	Univariate analysis		Multivariate analysis	
	OR (95% CI)	*p*‐value	OR (95% CI)	*p*‐value
Age ≥70 years	5.040 (1.509, 16.833)	0.009*	2.687 (0.677,10.663)	0.160
Gender (male vs. female)	0.356 (0.095, 1.333)	0.125	0.432 (0.101, 1.856)	0.259
Study phase (1 vs. 2/3)	0.786 (0.233, 2.648)	0.697		
Relapsed/refractory	0.459 (0.110, 1.919)	0.282		
BMI ≥25 kg/m^2^	0.616 (0.184, 2.063)	0.432		
HB <12 g/L	1.403 (0.441, 4.463)	0.566		
Lymphocyte <800/mmc	1.536 (0.295, 7.990)	0.610		
NLR >3.5	1.510 (0.434, 5.257)	0.517		
Vaccination	3.929 (1.302, 11.855)	0.015*	2.181 (0.618, 7.699)	0.226
≥3 doses of vaccination	0.560 (0.067, 4.702)	0.593		
i‐BNHL versus other	0.076 (0010, 0.597)	0.014*	0.113 (0.013, 0.945)	0.044*
Advanced stage	0.316 (0.100, 1.002)	0.050		
IPI ≥2	1.034 (0.346, 3.090)	0.952		
Ever smoker	2.613 (0.890, 7.673)	0.081		
PTH CD20 antibody	1.031 (0.329, 3.233)	0.958		
Previous treatment line ≥3	2.167 (0.741, 6.335)	0.158		
CR/PR at COVID‐19	0.343 (0.116, 1.014)	0.053		
Comorbidity ≥1	3.229 (1.077, 9.685)	0.036*	2.216 (0.635, 7.737)	0.212
PI3Ki‐based therapy	0.374 (0.079, 1.759)	0.213		
Anti‐CD20 therapy	1.407 (0.406, 4.877)	0.590		
PTH bendamustine	0.456 (0.055, 3.769)	0.456		
PTH PD‐1/PD‐L1	0.816 (0.167, 3.990)	0.802		

Abbreviations: ASCT, autologous stem‐cell transplantation; BMI, body mass index; CART, chimeric antigen receptor T cell; CR, complete response; HB, hemoglobulin; i‐BNHL, indolent B‐cell non‐Hodgkin's lymphoma; IPI, international prognostic index; NLR, neutrophil‐to‐lymphocyte ratio; OR, odds ratio; PI3Ki, PI3K inhibitors; PR, partial response; PTH, previous treatment history.

* *p* < 0.05.

## 4 DISCUSSION

In the present study, we reported the outcome of COVID‐19 in lymphoma patients participating in registered clinical trials during the first wave of the Omicron outbreak in China. To the best of our knowledge, this is the largest series of Chinese lymphoma patients reported and the first study focusing on lymphoma patients participating in registered clinical trials so far. Clinical features of COVID‐19 reported in our cohort were similar to that of non‐HM patients, while the proportion of severe/critical COVID‐19 and the mortality rate were relatively higher than non‐HM patients. In addition, significant risk factors associated with the outcome of COVID‐19 were also identified. These findings might be helpful to health commissions in their decision‐making processes for lymphoma patients and physicians in their management of both clinical trials and lymphoma patients.

The clinical outcome of COVID‐19 in patients with solid cancers and HMs has been explored in a series of studies in the early phase of the SARS‐CoV‐2 pandemic period, indicating that those with malignancies were at increased risk of severe COVID‐19 outcomes.^19^ COVID‐19 disproportionately impacted lymphoma patients, with a relatively high mortality rate (13%–44%).^9^, ^16^, ^20^, ^21^ The subsequent Omicron variants of SARS‐CoV‐2, which harbor multiple novel spike protein mutations, were found to have increased infectivity compared with the delta variants but decreased hospitalization rate and mortality.^22^ The EPICOVIDEHA survey reported the clinical outcome of 593 HM patients infected with Omicron, demonstrating a mortality rate of 16.7% in hospitalized patients.^23^ However, rare studies have investigated the outcome of lymphoma patients infected with the Omicron variant. In the latest Omicron wave in China, Zhu et al. conducted a retrospective study to assess the outcomes of HM patients with COVID‐19. In that study, only 52 lymphoma patients were included with two severe/critical cases of COVID‐19 (2/51, 3.9%), while no death was observed.^14^ However, the impact of relevant data, such as pathological subtypes of lymphoma and different therapeutic approaches, was not discussed explicitly in that study.

Registered clinical trials are essential therapy options for lymphoma patients and are imperative to advancing lymphoma care, which were also inevitably disrupted during the COVID‐19 pandemic. Given that patients participating in registered clinical trials had good treatment compliance and detailed medical records, the current study could comprehensively explore the association between clinical variables and the outcome of COVID‐19. The spectrum of COVID‐19‐related symptoms in our cohort was similar to that of non‐cancer population, whereas our patients spent more time clearing the virus and recovering from COVID‐19‐related symptoms. Compared with the low mortality rate of 0.09% in non‐cancer population with the Omicron variant,^22^ the mortality was much higher in our entire cohort (2.73%) and in the hospitalized group (18.8%). Of note, one patient's death was due to progressive lymphoma but not COVID‐19. Severe/critical COVID‐19 was observed in 5.5% of our entire cohort and 37.5% of the hospitalized group, while a relatively lower proportion of severe/critical COVID‐19 (3.8%) was observed in lymphoma patients in Zhu's study.^14^ On the contrary, severe/critical COVID‐19 was only observed in 0.05% of non‐cancer patients based on published Omicron infection data,^22^ implying that the Omicron variant remained a significant challenge to lymphoma patients, especially in hospitalized patients and severe/critical diseases.

We selected the severity of COVID‐19 as the primary outcome in this study, and three significant risk factors were identified, including age ≥70 years, comorbidities, and PI3Ki‐containing regimens. Most previous studies^14^, ^16^, ^21^ have revealed that elderly HM patients were more likely to have unfavorable outcomes of COVID‐19, including severe/critical illness, prolonged in‐hospital stay, and death. In our study, more severe/critical (4/42, 9.5% vs. 5.5% overall) COVID‐19 cases were observed in patients with comorbidities (≥1). Similar findings were reported by Duléry^16^ and Zhu et al.^14^ Together with our data, these findings highlight that more attention should be paid to elderly lymphoma patients and frail patients with comorbidities diagnosed with COVID‐19 in registered clinical trials.

Different therapeutic approaches to lymphoma were also explored in our study. Previous anti‐CD20 antibodies^16^ and bendamustine treatment^17^ were reported in lymphoma patients in a subset of studies,^21^, ^24^ while their impact on COVID‐19 outcome remained controversial; however, neither reached statistical significance in our study. In contrast to previous studies, 89.1% of patients had relapsed/refractory diseases and the majority received heterogenous treatments of multiple novel agents influencing immune function in our study, which could partly neutralize the adverse impact of previous anti‐CD20 or bendamustine on COVID‐19. Nevertheless, the PI3Ki‐containing regimen was significantly associated with the severity of COVID‐19. Various PI3K inhibitors have been evaluated for lymphoma patients.^25^ Besides PI3Ki on the market,^26^, ^27^ many PI3K is are in ongoing trials in China. Despite the encouraging efficacy of PI3Ki, high infection rates were observed, including sepsis and pneumonia.^26^, ^27^ The severe and fatal infections could be partly attributed to T‐cell immune response impairment by PI3Ki.^28^ Our findings also underscore that lymphoma patients with COVID‐19 who receive PI3Ki should be early intervened and given effective anti‐viral treatment.

Vaccines of COVID‐19 have been proven to be safe and effective for preventing infection or attenuating disease severity and are also recommended to cancer patients. However, the concerns of vaccines included cancer progression, potential long‐term toxicity, or interfere with treatment resulted in a low acceptance of the vaccine in cancer patients in China.^29^, ^30^, ^31^ In our study, only 34.5% of patients were vaccinated against COVID‐19 and 10% of patients had received the third dose of vaccines. The lower vaccine serologic response was reported in HM patients, particularly those with lymphoid cancers and/or receiving anti‐CD20 therapy with impaired immune function^32^, ^33^, ^34^, ^35^; thus, the protective effect of vaccine might be limited in these patients.^14^, ^23^ Similar data on COVID‐19 severity among the unvaccinated and the vaccinated were speculated in our study, and the third vaccination dose did not show protective effect. Possible explanations might be the lower proportion of vaccinated cases, inactivated vaccines, immunosuppressive novel treatments. This aspect should be cautiously speculated. Adapted vaccination schedules for lymphoma patients, especially when exposed to novel immunosuppressive agents with complex mechanisms and potential unknown toxicity, are urgently needed in the era of Omicron.

Hospitalization was generally defined as one of the most severe outcomes of COVID‐19 in most previous studies, which was also selected as the secondary outcome in our study. Compared with the severity of COVID‐19 evaluated by investigators, the hospitalization for COVID‐19 was affected by many non‐objective factors, including the shortage of medical capability during the pandemic of Omicron in China. Our data revealed that patients with i‐BNHLs were less likely to be hospitalized for COVID‐19.

In contrast to most previous studies, the current study focused notably on lymphoma patients participating in registered clinical trials, 89.1% of whom relapsed after standard frontline therapy or developed refractory disease to prior treatment, while the distribution of lymphoma subtype was generally similar to previous data.^21^ Given the scarcity of data regarding this particular population, our results might provide valuable evidence to improve patient clinical trial access during the pandemic of COVID‐19. However, unfit patients, super‐elderly (>80 years) patients, patients with a short survival expectance and poor performance status who were ineligible for registered clinical trials could not be discussed in our study. Other limitations were the retrospective nature of this study, the lack of high‐throughput sequencing data on COVID‐19, and the systematic monitoring of immune responses. Long‐term follow‐up and sequential monitoring of immune responses are warranted.

In conclusion, this real‐world study reported ameliorative outcomes of COVID‐19 in lymphoma patients participating in clinical trials during the latest pandemic of the Omicron variant in China, with a relatively low severe/critical illness rate and mortality. Advanced age (≥70 years), comorbidities, and PI3Ki‐containing regimen were significantly associated with the severity of COVID‐19 in this particular population, which should be emphasized in the process management of registered clinical trials. Our findings provided valuable data to aid clinical researchers with managing lymphoma patients participating in registered clinical trials during the ongoing pandemic of the Omicron variant.

## AUTHOR CONTRIBUTIONS

**Peng Sun:** Conceptualization (lead); data curation (lead); formal analysis (lead); methodology (equal); resources (equal); software (equal); writing – original draft (lead). **Hang Yang:** Conceptualization (equal); data curation (equal). **Baitian Zhao:** Conceptualization (equal); data curation (equal); formal analysis (equal); methodology (equal). **Yu Wang:** Conceptualization (equal); investigation (equal). **Man Nie:** Formal analysis (equal); software (equal); writing – original draft (equal). **Kangming Huang:** Formal analysis (equal); software (equal). **Zhiming Li:** Conceptualization (lead); funding acquisition (equal); supervision (lead); writing – review and editing (equal).

## FUNDING INFORMATION

This work was supported by grants from the National Science and Technology Major Project (no. 2018ZX09734003) and the National Natural Science Foundation of China (nos. 81872902, 82073917, 82103579, and 82104273).

## CONFLICT OF INTEREST STATEMENT

All authors declare no conflicts of interest.

## Supporting information


Data S1:

## Data Availability

We have uploaded the essential raw data onto the Research Data Deposit (RDDA2023450151) public platform (https://www.researchdata.org.cn).
