# Supplementary material for: Outcomes of COVID‐19 in patients with lymphomas participating in registered clinical trials: A real‐world study from China in the Omicron outbreak era
Source: Cancer Med. 2023 Nov 27;12(23):21148–58. doi: 10.1002/cam4.6678 (PMC10726839; doi:10.1002/cam4.6678)

Table S1 Description of two patients who died during hospitalization for COVID-19

| Age (years) | Gender | Comorbidity | Lymphoma subtype | Last treatment of lymphoma | Study phase | Interval between COVID-19 and death (days) | Lymphoma status | COVID-19  treatment |
| --- | --- | --- | --- | --- | --- | --- | --- | --- |
| 50 | Male | None | PTCL, NOS | PI3Ki from 2022/11/09 | 1 | 46 | Not evaluated | Azvudine and  antibiotics |
| 32 | Male | Hepatitis B virus  infection | DLBCL | BTKi and PI3Ki from  2022/11/02 | 1 | 73 | SD | Azvudine and  antibiotics |

DLBCL, diffuse large B cell lymphoma. PTCL,NOS, peripheral T cell lymphoma, Not Otherwise Specified. PI3Ki, PI3K inhibitors. BTKi, Bruton’s tyrosine kinase inhibitors. SD, stable disease.

Figure S1 Study profiles


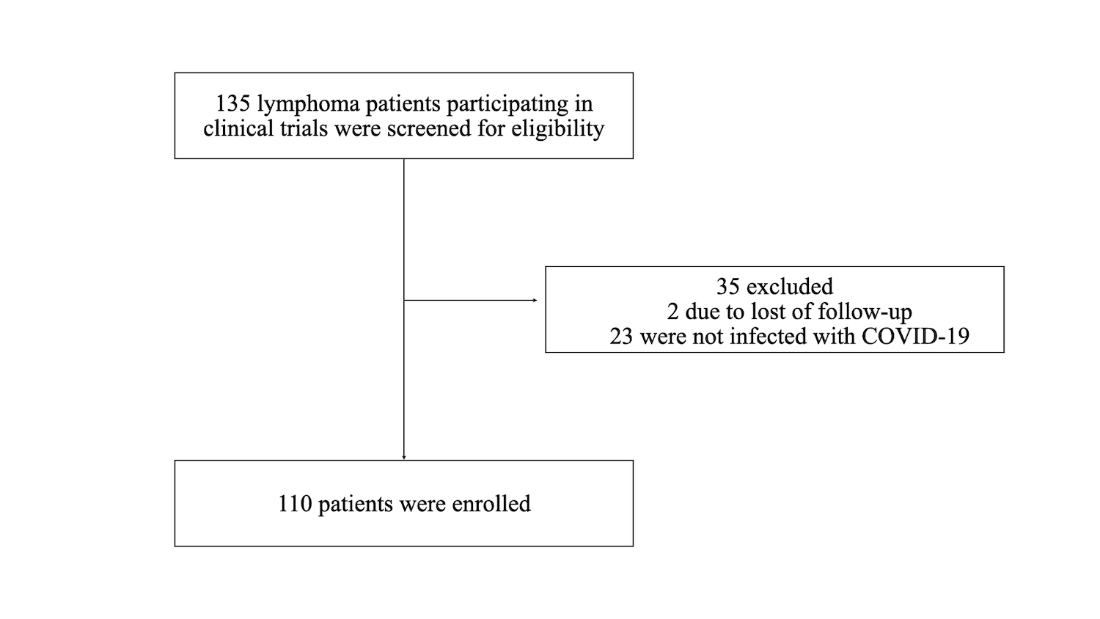

Supplement: Supplementary file 1 — Data S1: [file CAM4-12-21148-s001.docx]
